# Evaluation of Hepatitis in Pediatric Patients With Presumed Nonalcoholic Fatty Liver Disease

**DOI:** 10.1097/PG9.0000000000000181

**Published:** 2022-03-09

**Authors:** Fat’hiya Al-Harthy, Neha Kamath, Lee Hill, Jelena Popov, Allison Bossert, Herbert Brill, Nikhil Pai

**Affiliations:** From the * Department of Pediatrics, Division of Gastroenterology and Nutrition, McMaster University, Hamilton, Canada; † Faculty of Science, University of Western Ontario, London, Canada; ‡ Department of Pediatrics, Division of Gastroenterology and Nutrition, McMaster University, Hamilton, Canada; § Department of Exercise Science and Sports Medicine, University of Cape Town, Cape Town, ZA; ∥ Department of Pediatrics, Division of Gastroenterology and Nutrition, McMaster University, Hamilton, Canada; ¶ College of Medicine and Health, University College Cork, Cork, Republic of Ireland; # Pediatric Gastroenterology and Nutrition, Hamilton Health Sciences Corporation (McMaster Children’s Hospital), Hamilton, Canada; ** Department of Pediatrics, Division of Gastroenterology and Nutrition, McMaster University, Hamilton, Canada; †† Department of Pediatrics, Division of Gastroenterology and Nutrition, McMaster University, Hamilton, Canada; ‡‡ Department of Medicine, Farncombe Family Digestive Diseases Research Institute, McMaster University, Hamilton, Canada.

**Keywords:** NAFLD, guidelines, chronic hepatitis, testing, pediatric

## Abstract

In 2017, the North American Society of Pediatric Gastroenterology, Hepatology and Nutrition published clinical practice guidelines for the assessment and diagnosis of nonalcoholic fatty liver disease (NAFLD). We determined how frequently these investigations suggest an alternate etiology for chronic hepatitis in 8- to 17-year-old patients with body mass index >85%, elevated alanine aminotransferase and radiographic steatosis, and rates of adherence to 2017 guidelines.

**Methods:**

We conducted a retrospective chart review of patients presenting to McMaster Children’s Hospital from 2017–2020 for evaluation of suspected NAFLD. Bloodwork was reviewed.

**Results:**

Ninety-five patients met inclusion criteria. Abnormal bloodwork that required further testing was found in 28.4%; a different chronic liver disease was ultimately diagnosed in 11.6%. Only 9.5% received comprehensive, additional bloodwork for other causes of liver disease.

**Conclusion:**

A high proportion of patients evaluated for suspected NAFLD had bloodwork possibly suggesting an alternate diagnosis. Comprehensive testing was infrequently performed. These results reinforce the importance of maintaining a differential diagnosis among children presumed to have NAFLD.

**What Is Known**

- Nonalcoholic fatty liver disease (NAFLD) is one of the most common pediatric liver diseases.
- In 2017, the North American Society of Pediatric Gastroenterology, Hepatology and Nutrition published clinical practice guidelines on the assessment and management of NAFLD.

**What Is New**

- Our retrospective chart review found 28.4% of patients with clinical, laboratory, and radiographic signs of NAFLD had abnormal results from additional testing.
- 11.6% were diagnosed with a chronic liver disease resulting from this testing.
- Only 9.5% of patients had comprehensive, additional testing performed per the 2017 North American Society of Pediatric Gastroenterology, Hepatology and Nutrition guidelines.

Nonalcoholic fatty liver disease (NAFLD) is a chronic liver condition resulting from excessive fat accumulation in the liver. The prevalence of pediatric NAFLD has increased in parallel with the global rise in obesity over the past 30 years.^1^ NAFLD is now the most common cause for liver transplantation among young adults.^2^ Thirty-four percent of overweight and obese children and adolescents are estimated to have NAFLD, and these global rates are shared across Canada.^1,3–5^ In 2017, the North American Society of Pediatric Gastroenterology, Hepatology and Nutrition (NASPGHAN) published clinical practice guidelines on the assessment and management of NAFLD.^6^ While children with a body mass index >85% for age may be suspected of having NAFLD with elevated alanine aminotransferase (ALT) and radiographic features of hepatic steatosis, these features are also common across other chronic liver diseases. A diagnosis of NAFLD may only be made after the exclusion of other underlying diagnoses including infectious hepatitis, autoimmune hepatitis (AIH), celiac disease, and others.

The prevalence of these liver diseases varies widely in children. Hepatitis A infection has an estimated prevalence of 0.4 cases per 100 000,^7^ chronic Hepatitis B virus infection <1%,^8^ and Hepatitis C between 0.3% and 9.0%.^9^ The most common form of childhood-onset hypothyroidism, autoimmune thyroiditis, is estimated in 6.3%;^10^ AIH in 2.4 to 9.9 per 100 000;^11^ Wilson’s disease, 1 per 30,000; celiac disease, 300 to 1300 per 100 000;^12^ alpha-1-antitrypsin (A1AT) deficiency, 1 per 5000 to 7000;^13^ and lysosomal acid lipase (LAL) deficiency, 1 per 40 000.^14^

Few studies have assessed the prevalence of these chronic liver diseases in patients who have had suggested testing for NAFLD. A single-center retrospective study from 2013 evaluated 347 overweight or obese children referred to a pediatric gastroenterology clinic with ALT greater than 2× the upper limit of normal (ULN).^15^ 17.6% of patients were found to have a liver disease other than NAFLD, of which AIH was most common.^6^ A more recent multicenter, retrospective cohort study by Yodoshi et al from 2021 assessed 900 children with obesity. Only 2% were found to have another cause of liver disease.^16^ While both studies reported comprehensive institutional testing protocols, data were collected before the publication of the 2017 NASPGHAN guidelines. A predefined algorithm to guide recommended testing may have further improved the diagnostic workup of these patients. While liver biopsy remains the gold standard for diagnosing, staging, and excluding causes of hepatitis, invasiveness and potential for side effects have limited its clinical utility.^6^ While noninvasive bloodwork is endorsed, testing costs, patient and parent compliance, and perceived lack of clinical yield of ancillary testing may affect adherence to these recommendations.^6,17^

## OBJECTIVES

The overall aim was to assess the value of providing a comprehensive workup for conditions that could lead to chronic hepatitis in children suspected of having NAFLD.

Our primary objectives were to determine (1) how frequently patients had abnormal bloodwork suggestive of an alternative condition causing chronic hepatitis based on investigations recommended in the 2017 NASPGHAN guidelines^6^ and (2) how frequently these recommended investigations were performed.

Our secondary objectives were to determine the associated costs of conducting the recommended investigations.

## METHODOLOGY

We conducted a single-center, retrospective chart review of patients who presented to the McMaster Children’s Hospital (Hamilton, Canada) from 2017 to 2020. Included patients were 8 to 17 years old at the time of data collection, body mass index for age >85th percentile, persistently (>3 months) elevated ALT more than twice the ULN (<12 years, ULN = 45 U/L; males, 13–18 years, ULN = 49 U/L; females, 13–18 years, ULN = 38 U/L), and had radiographic (ultrasound, computed tomography, and MRI) features of hepatic steatosis. Patients with a preexisting diagnosis of chronic liver disease or actively taking medications associated with liver toxicity were excluded. Patients were identified through electronic medical records (Epic Systems Corporation), using an electronic reporting application designed with study inclusion and exclusion criteria.^18^ Study investigators additionally reviewed individual patient clinic lists to further verify that eligible patients were not omitted.

We reviewed bloodwork results recommended by the 2017 NASPGHAN Clinical Practice Guidelines: infectious hepatitis serologies (Hepatitis A virus IgM, Hepatitis B surface antigen, anti–Hepatitis C virus), thyroid studies (thyroid-stimulating hormone [TSH]), ceruloplasmin, A1AT, liver autoantibodies (antinuclear antibody; anti-smooth muscle antibody; liver kidney microsome type 1 antibody), tissue transglutaminase IgA (TTG-IgA), total IgA, total IgG, and LAL blood spot.^6^ We included bloodwork that had been measured at any point after the ALT became elevated >2× ULN. We assessed whether these recommended investigations were sent and estimated costs of performing investigations using data on laboratory testing costs through the Hamilton Regional Laboratory Medicine Program.^19^

Descriptive analyses were performed on all data. Frequencies were expressed as counts and percentages for all categorical variables. Ninety-five percent confidence intervals were reported for estimated percentages. Ethics approval was granted by the Hamilton Integrated Research Ethics Board (project number 5885-C).

## RESULTS

**FIGURE 1.**
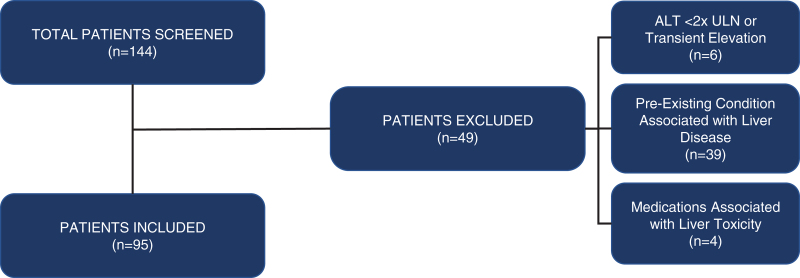
Results of patient flow and reasons for study exclusion. ALT = alanine aminotransferase; ULN = upper limit of normal.

One hundred forty-four patients met initial screening criteria. Forty-nine patients were excluded on manual review by investigators (F.A.-H. and N.P.) due to: transient ALT elevation with successive ALTs within normal range or ALT <2× ULN (n = 6), concomitant medications associated with liver toxicity (n = 4), or preexisting conditions associated with liver disease (n = 39; Fig. 1). Ninety-five patients were included. All patients had a preliminary diagnosis of NAFLD and were followed in pediatric outpatient clinics.

Twenty-seven (28.4%) patients had abnormal bloodwork: TSH (6, 10.3%), infectious hepatitis serologies (4, 11.7%), AIH antibodies (7, 4.2%), celiac serologies (6, 11.5%), A1AT (2, 4.0%), and quantitative immunoglobulins (2, 3.0%; Table 1).

**TABLE 1.** Adherence to testing among total population, results of recommended bloodwork for nonalcoholic fatty liver disease, and associated costs of assays

Test	Total* N = 95, n (%)	Abnormal Bloodwork,† n (%)	Abnormal Bloodwork Resulting in Diagnoses,‡ n (%)	Estimated Abnormal Results, n (95% CI)§	Cost (CAD$)∥
HepC Ab	44 (46.3)	1 (2.3)	1 (2.3)	2.2 (2.1–2.3)	15.00
HepA IgM	18 (18.9)	1 (5.6)	1 (5.6)	5.3 (5.2–5.5)	15.00
HepBS Ag	51 (53.7)	2 (3.9)	1 (2.0)	3.7 (3.6–3.8)	15.00
LAL	2 (2.1)	0 (0)	0	0	75.00
A1AT	50 (52.6)	2 (4.0)	1 (2.0)	3.8 (3.7–4.0)	150.00
ANA	60 (63.2)	7 (11.7)	1 (1.7)	11.1 (11.0–11.2)	22.75
ASMA	54 (56.8)	0 (0)	0	0	25.00
anti–LKM-1	53 (55.8)	0 (0)	0	0	27.75
Ceruloplasmin	52 (54.7)	0 (0)	0	0	6.30
IgG	65 (68.4)	1 (1.5)¶	0	1.5 (1.4–1.6)	2.50
IgA	65 (68.4)	1 (1.5)#	0	1.5 (1.4–1.6)	2.50
TTG-IgA	52 (54.7)	6 (11.5)	4 (7.7)	11.0 (10.9–11.2)	31.50
TSH	58 (61.1)	6 (10.3)	2 (3.4)	9.8 (9.7–10.0)	9.00

A1AT = alpha 1 antitrypsin; AMA = anti-mitochondrial antibody; ANA = antinuclear antibody; anti-LKM-1 = liver kidney microsome type 1 antibody; ASMA = anti-smooth muscle antibody; CAD = Canadian dollar; CI, confidence interval; HepA IgM = hepatitis A immunoglobulin M; HepBS Ag = hepatitis B surface antigen; HepC Ab = hepatitis C antibody; LAL = lysosomal acid lipase; TSH = thyroid-stimulating hormone; TTG-IgA = tissue transglutaminase immunoglobulin A.

*Patients who had testing performed across total study population.

†Patients with positive results among those who were tested.

‡Patients with diagnoses pending not included.

§Number of patients estimated to have positive results if testing was performed across the total study population.

∥Charges for phlebotomist and laboratory technician time not included.

¶IgG greater than the upper limit of normal for age.

#IgA less than the lower limit of normal for age.

Eleven (11.6%) patients were ultimately diagnosed with a condition resulting from their abnormal bloodwork: infectious hepatitis (3, 9.8%), thyroid disease (2, 3.4%), celiac disease (4, 7.7%), AIH (1, 1.7%; diagnosis based on liver biopsy), and A1AT deficiency (1, 2.0%).

Nine (9.5%) patients had >90% of recommended additional testing performed. Antinuclear antibody was measured most frequently (60, 63.2%), followed by TSH (58, 61.1%) and anti-smooth muscle antibody (54, 56.8%). LAL testing was performed least frequently (2, 2.1%), followed by Hepatitis A virus IgM (18, 18.9%; Table 1).

Seventy-three (76.8%) patients had suggested testing performed in our Pediatric Gastroenterology and Hepatology clinic, 14 (14.7%) were assessed through the Children’s Exercise and Nutrition Clinic, and 8 (8.4%) by other pediatric subspecialty services.

Unit testing cost of performing all recommended bloodwork was: $397.30 CAD (Table 1).^6,20^ Charges for phlebotomist and laboratory technician time were not included.

## DISCUSSION

The clinical and public health costs of NAFLD are significant, and its increasing prevalence in pediatric populations has long-term implications.^2,4,21,22^ Primary care clinicians and pediatric subspecialists are recognizing this by screening for NAFLD in overweight and obese patients; however, we need to remain focused on the differential diagnosis for chronic hepatitis. Our study identified low rates (9.5%) of comprehensive, additional testing for other causes of chronic liver disease. The consequences of false attribution of hepatitis to NAFLD can have important implications, particularly in children who may carry diagnostic labels unchecked through the life span.^23,24^ LAL testing was performed least frequently (2.1%). This may be particularly noteworthy, as recent literature has suggested reduced levels of LAL may be a modulator of susceptibility to NAFLD.^25^

We found high rates (27, 28.4%) of abnormal bloodwork that required further assessment for associated causes of chronic liver disease. Forty percent of these patients were ultimately diagnosed with a non-NAFLD, chronic liver disease consistent with their abnormal bloodwork. Our data emphasize the importance of conducting recommended testing. Notably, these figures were only based on testing that was performed. Many patients in our sample (86, 90.5%) had incomplete testing, and 20 (21.0%) had no additional investigations beyond basic liver enzymes. We estimated rates of abnormal bloodwork if comprehensive testing for associated causes of chronic liver disease had been performed across the total study population (Table 1).

In our study, we assessed the completion of investigations as per the 2017 guideline recommendations for children suspected of having NAFLD.^6^ Numerous factors may affect the uptake of guidelines in clinical practice, including reliance on outdated order sets, individual clinician judgment, and lack of guideline dissemination particularly to non-gastroenterologists.^26,27^ Our study only extended to 2020, 3 years after publication. All patients in this study were assessed by hospital-affiliated pediatric subspecialists, and 76.8% of included patients were last seen by the pediatric gastroenterology service (14.7%, Children’s Exercise and Nutrition Clinic; 8.4%, other pediatric subspecialty services). Our data show that despite this, patients suspected to have NAFLD continue to not receive recommended, guideline-based testing. In real-world practice, patients are also being diagnosed with NAFLD by family physicians and community pediatricians. Assuming the uptake of evidence-based guidelines is even more inconsistent in community practice, and patients having bloodwork at community laboratories have to pay out-of-pocket expenses for certain investigations, our figures likely underestimate the gaps in pediatric NAFLD diagnosis and management.^28–30^ Further supports are needed to improve timely, evidence-based practice in the diagnosis of pediatric NAFLD across all pediatric subspecialty and primary care settings.

Two previous studies assessed the results of chronic hepatitis testing in children suspected of having NAFLD. A 2013 study by Schwimmer et al found 17.6% (61/347) referred to a pediatric gastroenterology clinic for evaluation of NAFLD were ultimately diagnosed with non-NAFLD conditions.^15^ A 2021 multicentre, retrospective cohort study by Yodoshi et al found only 2.1% (19/900) had another cause of liver disease.^16^ Neither study specified what percentage of patients received comprehensive testing for these conditions. In our study, 11.6% (11/95) received a diagnosis other than NAFLD. There are several important differences in our methodology that may explain our results. Our study only included patients after 2017, following the publication of NASPGHAN guidelines.^6^ This may have increased rates of broad diagnostic testing compared with these other studies that involved cohorts collected earlier. Our study was conducted in Ontario, Canada, where universal health insurance eliminates many socioeconomic barriers to care and diagnostic testing. The prevalence of celiac disease in our study (11.5% with elevated TTG-IgA; 7.7% ultimately diagnosed with celiac disease) was significantly higher than the rates reported by Yodoshi et al (0.4%)^16^ and Schwimmer et al (1.2%).^15^ Only half (54.7%) of our cohort had celiac testing even performed. We estimated 11.6% (11; 95% confidence interval, 10.9-11.2) would have had an abnormal TTG-IgA if screening had been performed across our total study population (Table 1). As a modifiable condition (gluten-free diet), TTG-IgA remains an important test to perform in all patients with chronic hepatitis. Rates of thyroid disease requiring treatment by an endocrinologist (2.1%), A1AT deficiency (1.1%), and liver biopsy-proven AIH (1.1%) were similar to Yodoshi et al, who reported thyroid disease in 1.7%, A1AT deficiency in 0.4%, and no cases of AIH.^16^ This is in contrast to Schwimmer et al, who found 4.3% of cases with AIH. These discrepancies have been suggested to potentially reflect differences in race and ethnicity. This warrants further investigation, given the importance of early identification and treatment of this condition. Across all 3 cohorts (n = 1342), none identified a patient with Wilson’s disease.

We estimated the costs of performing the recommended testing as $397.30 CAD. These were derived from regional laboratory billing estimates and may not be consistent with other laboratories, particularly where universal health insurance is not offered. Nevertheless, these costs were modest and contrast with higher costs of testing for other conditions with similar long-term morbidity.^31,32^

Limitations of this study include its retrospective, single-center design. Variation in physician resources and practice styles may have affected uptake of testing guidelines. Other centers may have different practice patterns, referral criteria, criteria to perform liver biopsy, or different performance characteristics of diagnostic assays. We did not assess results of liver biopsy in our cohort, which prevented us from describing patients who may have concomitant features of NAFLD and thyroid dysfunction, celiac disease, or A1AT deficiency, as found by Yodoshi et al.^16^ Patients referred for presumptive NAFLD to pediatric gastroenterology clinics may have had more comprehensive testing performed compared to those patients who were not referred, although in our cohort, 76.8% of patients were assessed by a pediatric gastroenterologist. One patient in our study had positive HepA IgM. We were unable to determine vaccination status in our patient.^33^ Finally, while we included cost data as a relative measure of costs associated with testing, our study was restricted to patients in Ontario, Canada. There is significant geographic variation in testing costs, health insurance subsidization, and socioeconomic factors that affect patients’ abilities to obtain proper diagnostic workup. Thus, our data reflect only part of the many financial implications that must be considered to extrapolate these data to other practice locations.

There are several opportunities for future work. A broader, prospective multicenter study would help determine whether our findings are representative of other centers’ experiences. An assessment of factors that influence uptake of clinical practice guidelines would be valuable, particularly among pediatric gastroenterologists. Finally, there is value in performing further qualitative assessments to complement these data. Multiple factors affect providers’ willingness to offer testing including patients’ socioeconomic circumstances and concerns about timeliness of obtaining results. There may also be fears of increasing nonadherence to lifestyle-based treatments by encouraging families to seek other reasons for their liver disease.^6,34^ Qualitative follow-up studies of both patients and providers would help discern these barriers.

Our study demonstrates the importance of performing broad diagnostic testing in pediatric patients suspected to have NAFLD. We must continue to be comprehensive in our approach to assessing other causes of chronic hepatitis in this patient population.

## References

[1] Anderson EL Howe LD Jones HE. The prevalence of non-alcoholic fatty liver disease in children and adolescents: a systematic review and meta-analysis. PLoS One. 2015;10:e0140908. 26512983 10.1371/journal.pone.0140908 PMC4626023

[2] Pais R Barritt AS 4th Calmus Y. NAFLD and liver transplantation: current burden and expected challenges. J Hepatol. 2016;65:1245–1257. 27486010 10.1016/j.jhep.2016.07.033 PMC5326676

[3] Yu EL Golshan S Harlow KE. Prevalence of nonalcoholic fatty liver disease in children with obesity. J Pediatr. 2019;207:64–70. 30559024 10.1016/j.jpeds.2018.11.021 PMC6440815

[4] Rao D Kropac E Do M Roberts K Jayaraman G. Status report - childhood overweight and obesity in Canada: an integrative assessment. Heal Promot Chronic Dis Prev Canada. 2017;37:87–93. 10.24095/hpcdp.37.3.04 PMC5602163 28273036

[5] Mohamed RZ Jalaludin MY Anuar Zaini A. Predictors of non-alcoholic fatty liver disease (NAFLD) among children with obesity. J Pediatr Endocrinol Metab. 2020;33:247–253. 31926095 10.1515/jpem-2019-0403

[6] Vos MB Abrams SH Barlow SE. NASPGHAN clinical practice guideline for the diagnosis and treatment of nonalcoholic fatty liver disease in children: recommendations from the expert committee on NAFLD (ECON) and the North American Society of Pediatric Gastroenterology, Hepatology and Nutrition (NASPGHAN). J Pediatr Gastroenterol Nutr. 2017;64:319–334. 28107283 10.1097/MPG.0000000000001482 PMC5413933

[7] Division of Viral Hepatitis. Hepatitis A: Hepatitis Surveillance Report. Atlanta, GA, GA; 2019. https://www.cdc.gov/hepatitis/statistics/2017surveillance/TablesFigures-HepA.htm#tabs-1-6.

[8] World Health Organization. Hepatitis B Fact Sheet. https://www.who.int/news-room/fact-sheets/detail/hepatitis-b. Published 2021. Accessed October 1, 2021.

[9] Centers for Disease Control and Prevention. Surveillance for Viral Hepatitis. Atlanta, GA, GA; 2018. https://www.cdc.gov/hepatitis/statistics/2016surveillance/index.htm#tabs-3-4.

[10] Hollowell JG Staehling NW Flanders WD. Serum TSH, T(4), and thyroid antibodies in the United States population (1988 to 1994): National Health and Nutrition Examination Survey (NHANES III). J Clin Endocrinol Metab. 2002;87:489–499. 11836274 10.1210/jcem.87.2.8182

[11] Mack CL Adams D Assis DN. Diagnosis and management of autoimmune hepatitis in adults and children: 2019 practice guidance and guidelines from the American Association for the Study of Liver Diseases. Hepatology. 2020;72:671–722. 31863477 10.1002/hep.31065

[12] Gujral N Freeman HJ Thomson AB. Celiac disease: prevalence, diagnosis, pathogenesis and treatment. World J Gastroenterol. 2012;18:6036–6059. 23155333 10.3748/wjg.v18.i42.6036 PMC3496881

[13] de Serres FJ Blanco I. Prevalence of α1-antitrypsin deficiency alleles PI*S and PI*Z worldwide and effective screening for each of the five phenotypic classes PI*MS, PI*MZ, PI*SS, PI*SZ, and PI*ZZ: a comprehensive review. Ther Adv Respir Dis. 2012;6:277–295. 22933512 10.1177/1753465812457113

[14] Carter A Brackley SM Gao J. The global prevalence and genetic spectrum of lysosomal acid lipase deficiency: a rare condition that mimics NAFLD. J Hepatol. 2019;70:142–150. 30315827 10.1016/j.jhep.2018.09.028

[15] Schwimmer JB Newton KP Awai HI. Paediatric gastroenterology evaluation of overweight and obese children referred from primary care for suspected non-alcoholic fatty liver disease. Aliment Pharmacol Ther. 2013;38:1267–1277. 24117728 10.1111/apt.12518 PMC3984047

[16] Yodoshi T Orkin S Arce-Clachar AC. Alternative etiologies of liver disease in children with suspected NAFLD. Pediatrics. 2021;147:e2020009829. 33785637 10.1542/peds.2020-009829 PMC8015155

[17] Koot BGP Nobili V. Screening for non-alcoholic fatty liver disease in children: do guidelines provide enough guidance? Obes Rev. 2017;18:1050–1060. 28544608 10.1111/obr.12556

[18] Epic Systems. https://www.epic.com/about. Published 2021. Accessed July 12, 2020.

[19] HHSC, HHS. Hamilton Regional Laboratory Medicine Program. Hamilton Health Sciences. https://www.hamiltonhealthsciences.ca/areas-of-care/services/laboratory/. Published 2020. Accessed July 12, 2020.

[20] Hamilton Regional Laboratory Medicine Program. Hamilton Regional Laboratory Medicine: COVID-19. https://ltig.hrlmp.ca/ViewTestHRLMP.aspx?testID=2156. Published 2020. Accessed July 12, 2020.

[21] Welsh JA Karpen S Vos MB. Increasing prevalence of nonalcoholic fatty liver disease among United States adolescents, 1988-1994 to 2007-2010. J Pediatr. 2013;162:496-500.e1. 23084707 10.1016/j.jpeds.2012.08.043 PMC3649872

[22] Younossi ZM Marchesini G Pinto-Cortez H. Epidemiology of nonalcoholic fatty liver disease and nonalcoholic steatohepatitis: implications for liver transplantation. Transplantation. 2019;103:22–27. 30335697 10.1097/TP.0000000000002484

[23] Schwimmer JB Dunn W Norman GJ. SAFETY study: alanine aminotransferase cutoff values are set too high for reliable detection of pediatric chronic liver disease. Gastroenterology. 2010;138:1357–64, 1364.e1. 20064512 10.1053/j.gastro.2009.12.052 PMC2846968

[24] Leonis MA Balistreri WF. Evaluation and management of end-stage liver disease in children. Gastroenterology. 2008;134:1741–1751. 18471551 10.1053/j.gastro.2008.02.029

[25] Francesco B Daniele P Domenico F. Reduced lysosomal acid lipase activity: a new marker of liver disease severity across the clinical continuum of non-alcoholic fatty liver disease? World J Gastroenterol. 2019;25:4172–4180. 31435171 10.3748/wjg.v25.i30.4172 PMC6700703

[26] Fontanesi J Messonnier M Hill L. A new model of adoption of clinical practice guidelines. J Public Health Manag Pract. 2007;13:605–611. 17984715 10.1097/01.PHH.0000296137.48929.a1

[27] Graham ID Harrison MB. Evaluation and adaptation of clinical practice guidelines. Evid Based Nurs. 2005;8:68–72. 16021701 10.1136/ebn.8.3.68

[28] Fischer F Lange K Klose K Greiner W Kraemer A. Barriers and strategies in guideline implementation—a scoping review. Healthcare. 2016;4:36. 10.3390/healthcare4030036 PMC5041037 27417624

[29] Lau R Stevenson F Ong BN. Achieving change in primary care–causes of the evidence to practice gap: systematic reviews of reviews. Implement Sci. 2016;11:40. 27001107 10.1186/s13012-016-0396-4 PMC4802575

[30] Knighton AJ McLaughlin M Blackburn R. Increasing adherence to evidence-based clinical practice. Qual Manag Health Care. 2019;28:65–67. 30586126 10.1097/QMH.0000000000000195

[31] Kuenzig ME Benchimol EI Lee L. The impact of inflammatory bowel disease in Canada 2018: direct costs and health services utilization. J Can Assoc Gastroenterol. 2019;2(suppl 1):S17–S33. 31294382 10.1093/jcag/gwy055 PMC6512251

[32] Park KT Ehrlich OG Allen JI. The cost of inflammatory bowel disease: an initiative from the Crohn’s & Colitis Foundation. Inflamm Bowel Dis. 2020;26:1–10. 31112238 10.1093/ibd/izz104 PMC7534391

[33] André F Van Damme P Safary A. Inactivated hepatitis A vaccine: immunogenicity, efficacy, safety and review of official recommendations for use. Expert Rev Vaccines. 2002;1:9–23. 12908508 10.1586/14760584.1.1.9

[34] Vittorio JM Lavine JE. Role of exercise in mitigating pediatric nonalcoholic fatty liver disease. Diabetes Care. 2020;43:280–282. 31959645 10.2337/dci19-0029

